# Microglia-triggered hypoexcitability plasticity of pyramidal neurons in the rat medial prefrontal cortex

**DOI:** 10.1016/j.crneur.2022.100028

**Published:** 2022-02-05

**Authors:** Yuki Yamawaki, Yayoi Wada, Sae Matsui, Gen Ohtsuki

**Affiliations:** a Department of Drug Discovery Medicine, Kyoto University Graduate School of Medicine, 53 Shogoin-Kawahara-cho, Kyoto, Japan; b Laboratory of Genetics, Kyoto University Graduate School of Biostudies, Yoshida-Shimoadachi-cho, Kyoto, Japan

**Keywords:** Intrinsic plasticity, LPS, L5 pyramidal neuron, mPFC, SK channel, Microglia

## Abstract

Lipopolysaccharide (LPS), an outer component of Gram-negative bacteria, induces a strong response of innate immunity via microglia, which triggers a modulation of the intrinsic excitability of neurons. However, it is unclear whether the modulation of neurophysiological properties is similar among neurons. Here, we found the hypoexcitability of layer 5 (L5) pyramidal neurons after exposure to LPS in the medial prefrontal cortex (mPFC) of juvenile rats. We recorded the firing frequency of L5 pyramidal neurons long-lastingly under in vitro whole-cell patch-clamp, and we found a reduction of the firing frequency after applying LPS. A decrease in the intrinsic excitability against LPS-exposure was also found in L2/3 pyramidal neurons but not in fast-spiking interneurons. The decrease in the excitability by immune-activation was underlain by increased activity of small-conductance Ca^2+^-activated K^+^ channels (SK channels) in the pyramidal neurons and tumor necrosis factor (TNF)-α released from microglia. We revealed that the reduction of the firing frequency of L5 pyramidal neurons was dependent on intraneuronal Ca^2+^ and PP2B. These results suggest the hypoexcitability of pyramidal neurons caused by the upregulation of SK channels via Ca^2+^-dependent phosphatase during acute inflammation in the mPFC. Such a mechanism is in contrast to that of cerebellar Purkinje cells, in which immune activation induces hyperexcitability via downregulation of SK channels. Further, a decrease in the frequency of spontaneous inhibitory synaptic transmission reflected network hypoactivity. Therefore, our results suggest that the directionality of the intrinsic plasticity by microglia is not consistent, depending on the brain region and the cell type.

## Highlights

• Microglia induce LTD of the intrinsic excitability of mPFC pyramidal neurons.
• The LTD of the intrinsic excitability is mediated by TNF-α and PP2B.
• Hypoexcitability during acute inflammation is via SK-channel upregulation.
• Directionality of immune-triggered plasticity is brain region- and cell type-dependent.

## Graphical abstract

**Figure:**
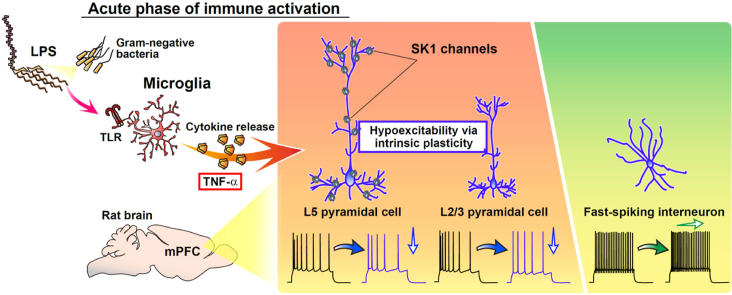

## Abbreviations

ACSF: artificial cerebrospinal fluid
AMPA receptor: α-amino-3-hydroxy-5-methyl-4-isoxazolepropionic acid receptor
AP: action potential
APV: _D_-(−)-2-Amino-5-phosphonopentanoic acid
ASD: Autism Spectrum Disorder
BAPTA: 1,2-Bis(2-aminophenoxy)ethane-N,N,N′,N′-tetraacetic acid
BK-channel: large conductance calcium-activated potassium channel
CaMKII: Ca^2+^/calmodulin-dependent protein kinase II
CCL2: chemokine (C–C motif) ligand 2
CK2: casein kinase 2
CNS: central nervous system
CSF-1R: colony-stimulating factor 1 receptor
DIC: differential interference contrast
FFT: fast Fourier transform
GABA: γ-aminobutyric acid
HCN channel: hyperpolarization-activated cyclic nucleotide-gated channel
IC50: half maximal inhibitory concentration
IL: interleukin
IP: intrinsic plasticity
L5: layer 5
LPS: lipopolysaccharide
LTD: long-term depression
LTP: long-term potentiation
MCP-1: monocyte chemoattractant protein-1
MIP: macrophage inflammatory protein
mIPSC: miniature inhibitory postsynaptic current
mPFC: medial prefrontal cortex
NBQX: 2,3-Dioxo-6-nitro-1,2,3,4-tetrahydrobenzo[f]quinoxaline-7-sulfonamide
NMDA receptor: N-methyl-D-aspartate receptor
n.s.: not significant
PAG: periaqueductal gray
PKA: protein kinase A
PKC: protein kinase C
PP2B: protein phosphatase 2B
S1: primary somatosensory cortex
SEM: standard error of the mean
sEPSC: spontaneous excitatory postsynaptic current
sIPSC: spontaneous inhibitory postsynaptic current
SK-channel: small-conductance Ca^2+^-activated K^+^ channel
TLR4: Toll-like receptor 4
TNF-α: tumor necrosis factor-α
TTX: tetrodotoxin
VTA: ventral tegmental area

## 1 Introduction

Recent accumulating evidence indicates that disturbance of the immune system is involved in the mechanisms of psychiatric disorders (Khandaker et al., 2015; Pape et al., 2019; Barichello et al., 2019; Segawa et al., 2021; Ozaki et al., 2021). Perhaps, excessive inflammatory cytokines, disruption of brain vasculature systems, and proliferation of activated immune cells in the parenchyma cause various symptoms of psychiatric disorders (Segawa et al., 2021). After the invasion of microbes and viruses into the central nervous system (CNS), the resident immune cells in the brain, microglia, are activated via the innate immune system. Transient exposure of brain slices to the Gram-negative bacterial endotoxin, lipopolysaccharide (LPS), activates microglia through Toll-like receptor 4 (TLR4). Exposure to LPS facilitates the vesicular release at hippocampal excitatory presynaptic terminals (Pascual et al., 2012). It was also shown that when oxygenation is impaired, exposure to LPS could depress the postsynaptic efficacy via glutamate receptors by the superoxide and nitric oxide production (Zhang et al., 2014). Such immune-triggered modulations of synaptic transmission have been reported in several studies (Pascual et al., 2012; Parkhurst et al., 2013; Zhan et al., 2014; Zhang et al., 2014; Yamamoto et al., 2019). In contrast, activated microglia also modulate non-synaptic membrane excitability in neurons in the neocortex, hippocampus, amygdala, and cerebellum (Gao et al., 2014; Klapal et al., 2016; Tzour et al., 2017; Duan et al., 2018; Yamamoto et al., 2019; Luo et al., 2019; Zheng et al., 2021). However, the cellular mechanism for inducing the long-term plasticity of intrinsic excitability is only known in a limited number of cell types. Importantly, the directionality of the increase or decrease in the excitability after exposure to LPS or immune activation is utterly unclear across brain regions, and it may depend on the brain regions and subtypes of neurons there. In the case of cerebellar Purkinje cells, the activation of microglia by exposure to LPS produces a long-term increase in the firing frequency of action potential, modulates membrane properties attributed to small conductance Ca^2+^-activated K^+^-channels (SK channels), and enhances the excitability of dendrites (Yamamoto et al., 2019). The increase in the frequency of spontaneous synaptic transmission also suggests the increase in the firing frequency of action potential of presynaptic neurons, cerebellar granule cells (Yamamoto et al., 2019). A proinflammatory cytokine released from microglia, tumor necrosis factor-α (TNF-α), is shown to trigger the intracellular cascade of Purkinje neurons, intensifying their excitability via Ca^2+^-dependent protein phosphatases. Immune-triggered hyperexcitability of Purkinje cells in the acute phase of microglial activation shares the mechanism with the induction of the excitability increase (Schonewille et al., 2010; Belmeguenai et al., 2010; Ohtsuki et al., 2012; Ohtsuki and Hansel, 2018; Yamamoto et al., 2019; Ohtsuki, 2020; Titley et al., 2020). However, it remains unknown whether other brain regions also have the identical mechanism between the immune-triggered modulation and the intrinsic plasticity of neuronal excitability. In excitatory pyramidal neurons, the secreted TNF-α activates downward signaling of TNF-receptors and the intraneuronal PP2B (Pribiag and Stellwagen, 2013), while the molecular mechanism that microglia modulate the neuronal intrinsic excitability is unclear. The cerebral cortex includes different cell types, and their physiological and morphological properties have been characterized (Radnikow and Feldmeyer, 2018). We consider it beneficial and informative to reveal the mechanism of the immune-triggered plasticity in the cerebrum towards understanding certain disease models. In this study, we investigate the plasticity in the medial prefrontal cortex (mPFC). We aimed to understand the mechanism of neuromodulation by microglia in infected brains, comparing the mechanism of immune-triggered plasticity of intrinsic excitability in the mPFC and cerebellum with the same methodology. Here, we chose different cell types of neurons from the mPFC, and we monitored their modulation of action potential firing against the immune challenge.

Non-synaptic plasticity of intrinsic excitability, such as the potentiation of spike firing, is distinct from the plasticity of synaptic transmission. Plasticity of the intrinsic excitability is often called intrinsic plasticity (IP), which is underlain by the modulation of Ca^2+^-activated and voltage-gated ion-channels that determines the membrane properties: *i.e.*, intrinsic excitability. In the cerebellar Purkinje cells, the IP has been originally indicated as long-term potentiation (LTP) of intrinsic excitability (Belmeguenai et al., 2010; Schreurs et al., 1991), whereas a recent study has revealed the induction of a form of long-term depression (LTD) (Shim et al., 2017). The IP has been studied in the different types of neurons not only in the cerebellum but also in the other brain regions: the neocortex, hippocampus, and brain stem. Many types of ion channels are involved in its expression. For example, sensory deprivation from whisker trimming alters neuronal firing properties through the downregulation of dendritic hyperpolarization-activated cyclic nucleotide-gated (HCN) channels in the barrel cortex (Feldman and Brecht, 2005; Breton and Stuart, 2009; LeMessurier and Feldman, 2018). *In vivo* whole-cell patch-clamp recording from layer 5 (L5) pyramidal neurons of the barrel cortex demonstrated the bidirectional induction of LTP and LTD of intrinsic excitability of living animals (Mahon and Charpier, 2012). There are multiple ways to modulate the intrinsic excitability of neurons. Ion-channels such as SK-, BK-type Ca^2+^-activated K^+^-channels, A-type K^+^ channels, and HCN channels, at least, are involved in long-lasting changes of the function (Ohtsuki et al., 2020). Among them, the implication of SK- and BK-channels in the plasticity of intrinsic excitability has been studied for decades (Daoudal et al., 2002; Daoudal and Debanne, 2003; Campanac et al., 2008; Adelman et al., 2012; Adelman, 2016; Fernández-Fernández and Lamas, 2021). Bock et al. (2019) suggested that the impact of the SK channel is different between soma and dendrites of neocortical L5 pyramidal neurons due to dendritic calcium spikes via activation of R-type calcium channels. In the neocortical L5 pyramidal cells, BK-type channels also regulate the burst firing of action potentials (Bock and Stuart, 2016). In the cerebellum, Golgi cells and vestibular nuclei neurons are shown to be BK-type-dependent involving Ca^2+^/calmodulin-dependent protein kinase II (CaMKII) (Hull et al., 2013; Nelson et al., 2003, 2005). Purkinje cells are SK2-type-dependent through Ca^2+^-dependent phosphatases (*i.e.*, protein phosphatase 2B, PP2B) (Schonewille et al., 2010; Belmeguenai et al., 2010). It is noteworthy that the directionality of LTP (*i.e.*, IP) and LTD of intrinsic excitability of cerebellar Purkinje cells is determined by the activity of PP2B (Schonewille et al., 2010; Belmeguenai et al., 2010) and protein kinase C (PKC) (Shim et al., 2017), respectively. This bidirectionality is seemingly akin to that of the synaptic plasticity in this cell. On the other hand, the directionality of LTP or LTD of synaptic efficacy in the hippocampus and neocortex is determined by CaMKII and PKC or protein phosphatases, respectively (Bienenstock et al., 1982; Jörntell and Hansel, 2006). Both the duration and concentration of the Ca^2+^ influx resulting from the neuronal activity determine the activation of those Ca^2+^-dependent kinases and phosphatases in neurons, and thus the directionality of synaptic plasticity (Bienenstock et al., 1982; Jörntell and Hansel, 2006; Ohtsuki et al., 2009). Moreover, the directionality of synaptic plasticity is inverted in the cerebellar Purkinje cells compared to the hippocampal and neocortical pyramidal neurons (Jörntell and Hansel, 2006). However, it is yet unknown whether the immune-triggered plasticity of the intrinsic excitability is also an inverted relationship between the Purkinje cells and pyramidal cells. Therefore, we studied immune-triggered modulation of the intrinsic excitability and its directionality in the L5, L2/3 pyramidal neurons, and fast-spiking interneurons in the mPFC. Also, we asked for the molecular mechanism for the intrinsic plasticity in L5 pyramidal neurons and attempted to compare the mechanisms in the mPFC and the cerebellum.

## 2 Material and methods

### 2.1 Patch-clamp recordings

**Fig. 1:**
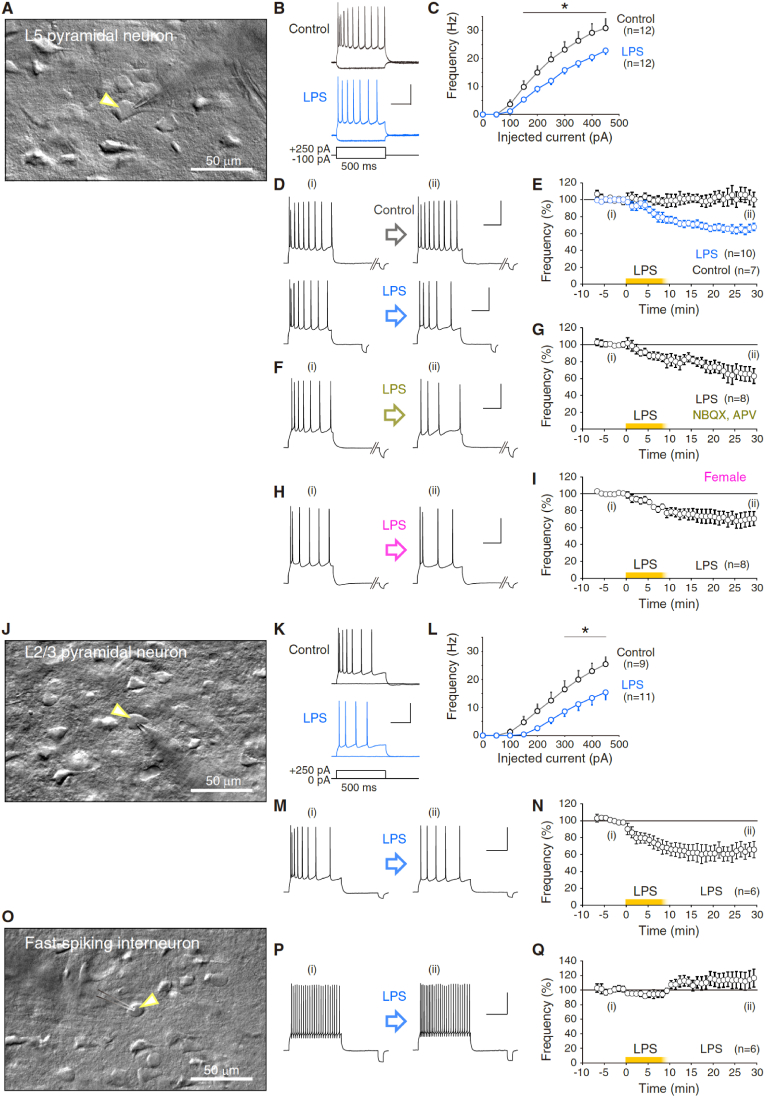
Exposure to bacterial endotoxin alters the excitability of excitatory pyramidal neurons in the mPFC. **(A**–**I)** Decrease in the firing frequency of medial prefrontal cortex (mPFC) layer 5 (L5) pyramidal neurons. (A) shows the representative DIC picture of the mPFC L5 pyramidal neuron during the patch-clamp experiment. The yellow arrowhead indicates the position of the cell under-recording. Representative traces of action potential (AP) firing of mPFC L5 pyramidal neurons before and after the exposure to lipopolysaccharide (LPS) (15–20 μg/mL) are shown in (B). The firing frequency of L5 pyramidal neurons of control and LPS-exposure experiments is shown in (C) (control, n = 12; LPS, n = 12. Asterisk indicates: p < 0.05 of Mann-Whitney *U* test). AP firing of mPFC pyramidal neurons in (C) was evoked by applying step pulses from 0 to 450 pA by 50 pA. **(D**–**I)** LPS perfusion induced a long-lasting decrease in the intrinsic excitability of mPFC L5 pyramidal cells. Representative AP firings before and after LPS exposure ((i) and (ii), respectively) are shown in (D). Experiments were done under suppression of fast GABAergic transmission by 100 μM picrotoxin. In (E), time courses of the firing-frequency changes are normalized at the average between −5 and −1 min (L5 pyramidal cells, control, n = 7; LPS, n = 10). In (F) and (G), the same long-term recordings were done under suppression of both fast GABAergic and glutamatergic synaptic transmission by picrotoxin, NBQX disodium salt (20 μM), and APV (50 μM) (n = 8). (H) and (I) show the long-term recordings from L5 pyramidal neurons of female rats (n = 8). (**J-N**) Decrease in the firing frequency of mPFC layer 2/3 (L2/3) pyramidal neurons. (J) shows the representative picture of the mPFC L2/3 pyramidal neurons. Representative traces of AP firing of mPFC L2/3 pyramidal neurons before and after exposure to LPS are shown in (K). Firing frequency of pyramidal neurons of control and LPS-exposure experiments is shown in (L) (control, n = 9; LPS, n = 11. Asterisk indicates: p < 0.05 of Mann-Whitney *U* test). AP firing of L2/3 pyramidal neurons in (L) was evoked by applying step pulses from 0 to 450 pA by 50 pA. (**M,N**) LPS perfusion induced a long-lasting decrease in the intrinsic excitability of mPFC L2/3 pyramidal cells. Representative AP firings are shown in (M). Experiments were done in the presence of picrotoxin. Normalized time course of the firing-frequency changes by LPS-exposure is shown in (N) (L2/3 pyramidal cells, n = 6). (**O-Q**) No substantial decrease in the firing frequency of mPFC fast-spiking neurons. (O) shows the representative picture of the mPFC fast-spiking neuron. (**P,Q**) Bath-application of LPS did not reduce the intrinsic excitability of fast-spiking neurons. Representative AP firings are shown in (P). Experiments were done in the presence of picrotoxin. Normalized time course of the firing-frequency changes by LPS-exposure is shown in (Q) (fast-spiking neurons, n = 6). All data are represented as mean ± SEM. Scales as 40 mV, and 200 ms. (For interpretation of the references to colour in this figure legend, the reader is referred to the Web version of this article.)

**Fig. 2:**
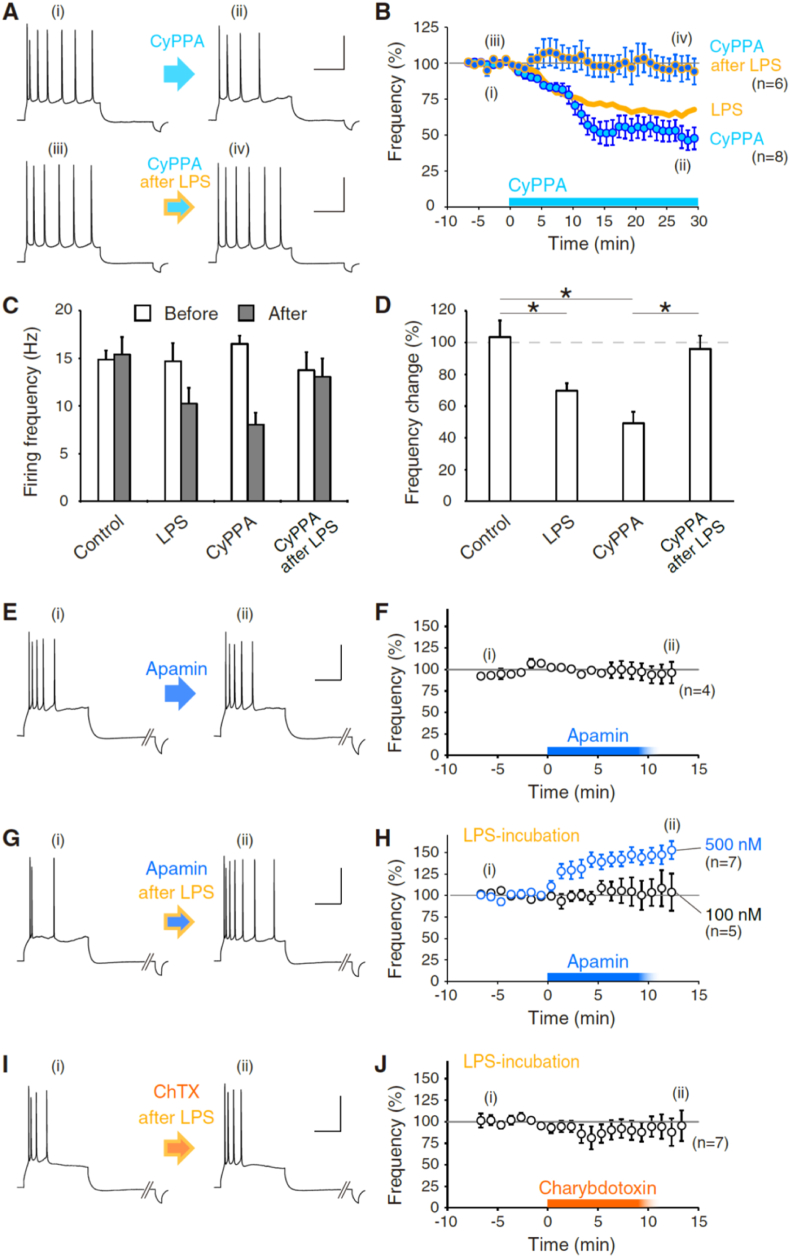
Upregulation of SK1-channel function underlies the LPS-induced firing frequency decrease of L5 pyramidal neurons. **(A,B)** Bath-application of an SK-channel agonist, CyPPA (10 μM), decreases the firing frequency of L5 pyramidal neurons. Pre-exposure to lipopolysaccharide (LPS) (15 μg/mL) for 10–20 min suppresses the decrease in firing frequency of pyramidal neurons by additional application of CyPPA. Representative action-potential (AP) firings of CyPPA application to naïve and LPS-treated L5 pyramidal neurons are shown in (A). Time courses of the normalized frequency of neurons applied CyPPA at 0 min after LPS-exposure and that without LPS-exposure are shown in (B) (CyPPA, n = 8; CyPPA after LPS, n = 6). For comparison, LPS-only data (15–20 μg/mL) is superimposed (yellow-orange line, identical to Fig. 1E). In the experiment of CyPPA application after LPS-exposure, the firing frequency before CyPPA application was adjusted to the level of control (7.7–20.0 Hz). **(C)** Summary bar graphs of firing frequency (Hz) before and after application of LPS, CyPPA, and CyPPA after LPS. **(D)** Summary bar graphs of firing-frequency changes by application of LPS, CyPPA, and CyPPA after LPS. CyPPA was less effective against neurons exposed to LPS beforehand. Asterisks indicate p < 0.01 of pairwise comparison. **(E,F)** Apamin perfusion did not change the AP firing of mPFC L5 pyramidal cells. Representative AP firings before and after 500 nM apamin application ((i) and (ii), respectively) are shown in (E). A time course of the normalized frequency of neurons applied apamin at 0 min is shown in (F) (n = 4). Experiments were done in the presence of 100 μM picrotoxin, 20 μM NBQX disodium salt, and 50 μM APV. **(G,H)** High concentration apamin-perfusion to LPS-preincubated mPFC increased AP firing of L5 pyramidal cells. Representative AP firings before and after 500 nM apamin-application ((i) and (ii), respectively) are shown in (G). Time courses of the normalized frequency of neurons applied two different concentrations of apamin at 0 min after LPS exposure are shown in (H) (Apamin of 500 nM after LPS, n = 7; Apamin of 100 nM after LPS, n = 5). Experiments were done in the presence of 100 μM picrotoxin, 20 μM NBQX disodium salt, and 50 μM APV. The increase in the AP firing in response to the application of 500 nM apamin after LPS-preexposure suggests the involvement of SK channel subtypes that are sensitive to high concentration apamin, *i.e.*, SK1 and SK3. **(I,J)** Charybdotoxin-perfusion to LPS-preincubated mPFC did not change AP firing of L5 pyramidal cells. Representative AP firings before and after 200-nM charybdotoxin application ((i) and (ii), respectively) are shown in (I). A time course of the normalized frequency is shown in (J) (Charybdotoxin after LPS, n = 7). Experiments were done in the presence of 100 μM picrotoxin, 20 μM NBQX disodium salt, and 50 μM APV. All the time courses of the firing frequency are normalized at the average between −5 and −1 min. All data are represented as mean ± SEM. Scales as 40 mV, and 200 ms. . (For interpretation of the references to colour in this figure legend, the reader is referred to the Web version of this article.)

*In vitro* patch-clamp recordings from cerebral L5, and L2/3 pyramidal neurons, and fast-spiking interneurons were obtained as described previously (Gill and Hansel, 2020) with a minor modification. Coronal sections of the cerebral prefrontal cortex (250 μm) at the position of +0.5- to +2.0-mm caudal from the bregma were prepared from male Sprague-Dawley rats (postnatal (P)24–33 days old). The slices were cut on a vibratome (Dosaka EM, Japan) using ceramic blades and kept in artificial cerebrospinal fluid (ACSF) containing the following (in mM): 124 NaCl, 5 KCl, 1.25 Na_2_HPO_4_, 2 MgSO_4_, 2 CaCl_2_, 26 NaHCO_3_, and 25 _D_-glucose, bubbled with 95% O_2_ and 5% CO_2_. During cutting, supplemental ingredients (5 mM Na-ascorbate, 2 mM thiourea, and 3 mM Na-pyruvate) were added to the chilled ACSF. After at least 1-h, the slices were transferred to a recording chamber superfused with ACSF at a near-physiological temperature (31–34 °C). The perfusate was supplemented with 100 μM picrotoxin to block GABA_A_ receptors in all the experiments except for Fig. 5. 20 μM NBQX disodium salt to block AMPA receptors, and 50 μM APV to block NMDA receptors were administered in the experiment of Fig. 1F and G, 2E–2J, 5, and S3. Patch-clamp recordings were performed under a × 40 water immersion objective lens equipped with a differential interference contrast (DIC) system (DS-Qi2, Nikon) mounted on a microscope (ECLIPSE FN1, Nikon), while recordings were performed in the voltage-clamp or current-clamp mode using an EPC-10 amplifier (HEKA Elektronik, Germany). Both membrane voltage and current were filtered at 2.9 kHz, digitized at 10 kHz, and acquired using PATCHMASTER software (HEKA Elektronik). Patch pipettes (borosilicate glass) were filled with K-gluconate solution which contains (in mM): 9 KCl, 10 KOH, 120 K-gluconate, 10 HEPES, 4 MgATP, 0.4 Na_3_GTP, 10 Na_2_-phosphocreatine, and 17.5 sucrose (pH 7.25 titrated with 1 M KOH). We confirmed in mPFC pyramidal neurons that the firing frequency attenuates in response to the repetitive test pulses without including the phosphocreatine in the pipette solution (n = 3; *data not shown*). Therefore, we used the pipette solution including Na_2_-phosphocreatine and confirmed the unchanged firing frequency of neurons during the recording of control condition (Fig. 1E) (Jung et al., 2001; Yu et al., 2009). Membrane voltage was offset for liquid junction potential (11.7 mV for K-gluconate solution; 12.5 mV for Cs-gluconate solution). The somatic patch electrodes had electrode resistances of 3–6 MΩ for L5 pyramidal cells, 4–6 MΩ for L2/3 pyramidal cells, and 4–10 MΩ for fast-spiking neurons. During the whole-cell patch-clamping, we compensated only the fast capacitance but not slow capacitance with the amplifier (HEKA EPC-10, and PATCHMASTER) to keep cells long-lastingly. The waveform of the action potential is obtained without compensation of slow capacitance, reflecting the cell membrane capacitance. After sealing and breaking the cell membrane under the voltage-clamp mode at −71.7 mV (L5 and L2/3 pyramidal cells, and fast-spiking neurons), bias currents (−100 – +50 pA for L5 pyramidal, L2/3 pyramidal, and fast-spiking neurons) were injected to stabilize the somatic membrane potential. Then, we switched the recording to current-clamp mode, and the membrane potential was held at approximately −72 mV. We applied the bridge balance under the current clamp (Park et al., 1983; Ito, 1957) to the experiments of Fig. 1F–I, 1K–1N, 1N, 1P, and 1Q, Fig. 2E–J, and Figure S3. A balanced bridge for the correction of membrane voltage errors is to inject current from the amplifier through the resistance of the pipette for measuring the true membrane voltage. To turn the bridge on using HEKA EPC-10 amplifier and PATCHMASTER, we set the time constant to the smallest value (*i.e.*, 10 μs) and set the percentage control to 92–100%. Then, the bridge compensates exactly the value of R-series. When we applied the bridge balance at a 10-μsec time constant from 0 to 100% to evaluate each parameter of action potential waveform, we noticed the decrease in peak voltage of 1.33 mV and that in the amplitude of 1.15 mV (0% vs. 100%), and no substantial difference in the input resistance in our experimental condition. Hence, we just have to note that we have given the overestimation of around 1-mV in the recordings of action potential peak and amplitude without the ideal bridge balance in the mPFC L5 pyramidal cells, which causes the approximately 1-mV errors (*data not shown*).

**Fig. 3:**
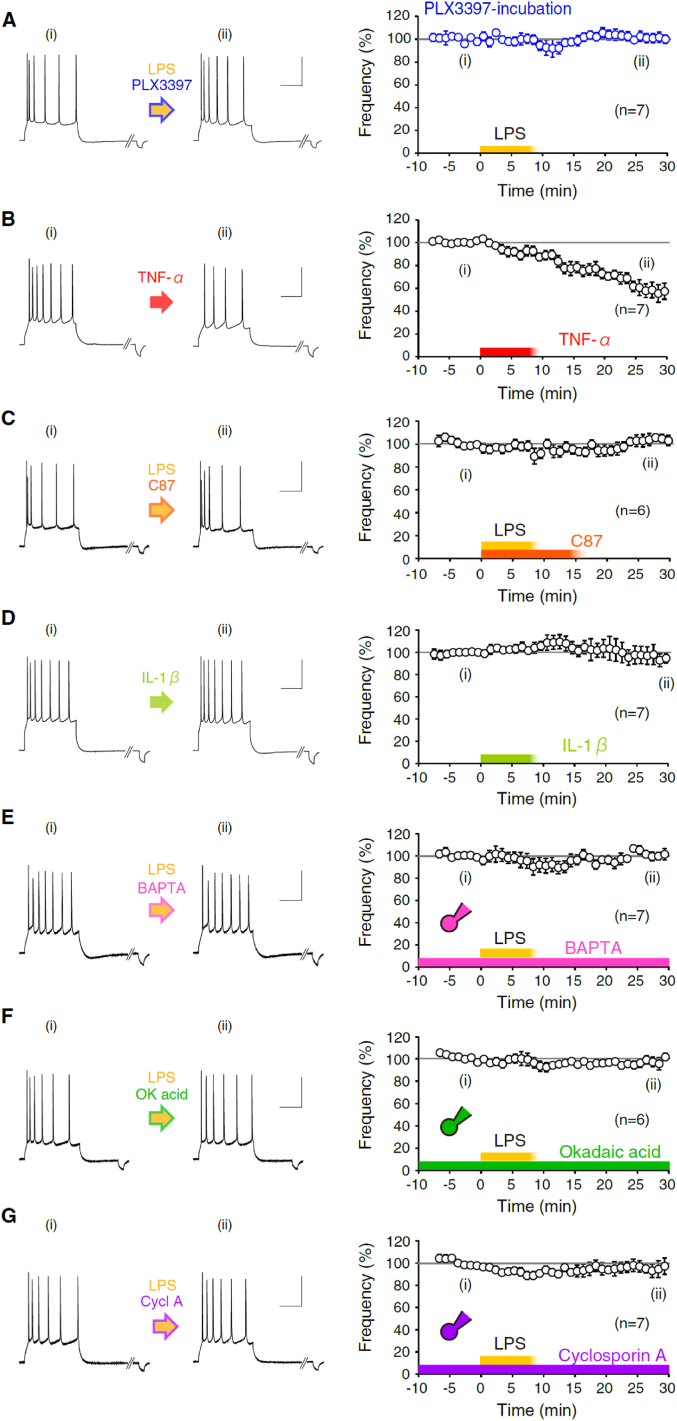
LPS-induced firing-frequency decrease of L5 pyramidal neurons is dependent on TNF-α release from microglia and intraneuronal PP2B signaling. **(A)** Action-potential (AP) firing of L5 pyramidal neurons in PLX3397 (Pexidartinib)-incubated mPFC slices. Representative AP firings before and after LPS exposure ((i) and (ii), respectively) are shown on the left. The time course of normalized firing frequency is shown in the right (n = 7). **(B)** Application of TNF-α (100–150 ng/mL) reduced the firing-frequency (n = 7). **(C)** Co-application of a blocker of TNF-α, C 87 (40 μM), and LPS blocked the induction of firing-frequency decrease (n = 6). **(D)** Application of IL-1β (3 nM) did not change the firing frequency (n = 7). **(E)** Intra-neuronal BAPTA (20 mM) impaired the plasticity of firing frequency by LPS perfusion (n = 7). We included the reagent in the pipette solution. **(F)** Intra-neuronal okadaic acid (300 nM) abolished the firing-frequency decrease plasticity by LPS perfusion (n = 6). **(G)** Intra-neuronal cyclosporin A (100 μM) inhibited the firing-frequency decrease plasticity by LPS perfusion (n = 7). In these graphs, all the time courses of the firing frequency normalized at the average between − 5 and −1 min are shown in (A–G). LPS or reagents application starts at 0 min for 8–15 min. All data are represented as mean ± SEM. Scales as 40 mV, and 200 ms.

For long-term recording (Fig. 1D–I, 1M, 1N, 1P, and 1Q, Fig. 2A, B, and 2E–2J, and Fig. 3), depolarizing current pulses (50–250 pA for the 500-ms duration) were applied every 20 s to the soma to evoke action potentials. In Figure S3, we applied the depolarizing test pulses at every minute. We compared firing frequency normalized by 5-min average before 0 min (*i.e.*, −5 to −1 min) to that of 25–30 min later. Input resistance was monitored by administering 50- or 100-pA hyperpolarizing pulses (for the 50-ms duration) following the depolarization. The input resistance under the current clamp is the sum of membrane resistance and series resistance. Data were discarded when the input resistance had changed more than 20% (Figures S1A & B). We have to note a limitation of our measurement of the input resistance under the current clamp to prove the thorough stability of recordings. It is because the measurement cannot observe each value simultaneously but just shows the stability of the summation. Membrane potential was kept at approximately from −68 to −76 mV for pyramidal cells and interneurons, within 5% changes throughout each experiment (Figures S1C & D). The rheobase for evoking action potential from the holding potential and the firing frequency during test period (: 5 to −1 min) for each data are shown in Figures S1E and S1F, respectively. In the mPFC slices, we recorded from L5 pyramidal neurons, L2/3 pyramidal neurons, and fast-spiking interneurons in the infralimbic and prelimbic cortex and identified individual neurons from their locations (: L5 pyramidal cells, 450–750 μm from the pia; L2/3 pyramidal cells, 200–430 μm from the pia), morphology (*e.g.*, L5 pyramidal cells are with a pyramidal shape of around 15 μm diameter; L2/3 pyramidal cells are with around 12 μm diameter; fast-spiking neurons are with a round shape of around 10 μm diameter) (see Fig. 1A, J, and 1O), passive membrane current (*e.g.*, low input resistance in L5 pyramidal cells, and high input resistance in interneurons), and firing properties (*e.g.*, pyramidal neurons evoke an initial burst spiking and following stable firing with a constant frequency with an obvious afterhyperpolarization of the action potentials). Pyramidal cells show a gradual increase in the action potential firing in response to gradually increasing step depolarization pulses, while fast-spiking interneurons show relatively all-or-none fashion. Reagents were applied to the bath chamber through a circulation system at a flow speed of 0.7–0.8 mL/min. In recordings with the reagent-containing internal solution, we waited for the invasion of a solution to dendritic processes more than 10 min after membrane break.

For the recording of spontaneous excitatory postsynaptic current (sEPSC), the membrane current was held at −71.7 mV using K-gluconate internal solution under picrotoxin, and the current was recorded for 1.5 s trials for at least 180 s in total. Serial resistance was monitored every 1.5 min and no substantial changes were found throughout the recordings. For the recording of spontaneous inhibitory postsynaptic current (sIPSC), and miniature IPSC (mIPSC) events, membrane voltage was held at +17.5 mV using Cs-gluconate internal solution (: 9 CsCl, 130 CsOH, 100 _D_-gluconic acid, 10 HEPES, 4 MgATP, 0.4 Na_3_GTP, 10 Na_2_-phosphocreatine, and 17.5 sucrose (pH 7.25 titrated with 2.6 M CsOH)) under NBQX disodium salt and APV. The current was recorded for 3.0 s trials for at least 360 s in total. mIPSC was recorded under suppression of the neuronal activity by tetrodotoxin (TTX) citrate (1 μM).

Reagents used in this study were purchased from the following sources and vendors, with the identifiers: Picrotoxin, Tocris/Bio-Techne, Cat# 1128, CAS: 124-87-8; _D_-APV, Cayman Chemical, Item# 14,539, CAS: 79,055-68-8; NBQX disodium salt, Tocris/Bio-Techne, Cat# 1044, CAS: 479,347-86-9; LPS from E. coli O111, Wako, Cat# 125–05201; CyPPA, Sigma-Aldrich, Cat# C5493, CAS: 73,029-73-9; apamin, Peptide Institute, Inc., Code# 4257-v, CAS: 24,345-16-2; charybdotoxin, Peptide Institute, Inc., Code# 4227-s, CAS: 95,751-30-7; PLX3397 (Pexidartinib), Cayman Chemical, Item# 18,271, CAS: 1,029,044-16-3; C 87, Tocris/Bio-Techne, Cat# 5484, CAS: 332,420-90-3; TNF-α, R&D Systems, Inc., Cat# 510-RT-010/CF; IL-1β, R&D Systems, Inc., Cat# 501-RL-010/CF; BAPTA, Sigma-Aldrich, Cat# A4926, CAS: 85,233-19-8; okadaic acid, AdipoGen, Cat# AG-CN2-0060, CAS: 78,111-17-8; cyclosporin A, Tocris/Bio-Techne, Cat# 1101, CAS: 59,865-13-3; TTX citrate, Tocris/Bio-Techne, Cat# 1069, CAS: 18,660-81-6.

### 2.2 Data analysis

Data were analyzed using a custom program written in MATLAB (Mathworks). For the analysis of sEPSC, sIPSC, and mIPSC events, an FFT bandpass (1–90 Hz) and a Savitzky-Golay filter (*smooth* function with Savitzky-Golay filter with 8 degrees of a polynomial model) were applied to the recorded currents. The method for the data analysis is already published in our previous studies (Yamamoto et al., 2019; Ohtsuki, 2020). In the event detection, the threshold for E/IPSCs applied to the filtered traces was set at 2 pA. Events were defined as those exceeding 3.5 times the standard deviation during the 4 ms pre-period, while the peak was detected within 10 ms after the initiation of an E/IPSC. Subsequently, we applied the *fminsearch* function to obtain the decay time of a single exponential. When the current trace at the decay period (limited to 19 ms) fit poorly, the data were excluded. The rise time was regarded as the period spanning between 10 and 90% of the change from the peak to the basement values.

[EPSC:] We took total 8576 sEPSC events from control L5 pyramidal neurons: 49–1578 events from 23 cells, total 9117 sEPSC events from LPS-exposed L5 pyramidal neurons: 252–3402 events from 14 cells, total 12,662 sEPSC events from TNF-α-exposed L5 pyramidal neurons: 122–3128 events from 15 cells, and total 9965 sEPSC events from IL-1β -exposed L5 pyramidal neurons: 92–1872 events from 9 cells, at least for 180 s. We took total 7803 sEPSC events from control L5 pyramidal neurons from females: 148–1478 events from 15 cells, and total 21,096 sEPSC events from LPS-exposed L5 pyramidal neurons from females: 454–2049 events from 16 cells. We took total 4146 sEPSC events from control L2/3 pyramidal neurons: 37–1754 events from 11 cells, and total 6972 sEPSC events from LPS-exposed L2/3 pyramidal neurons: 139–3108 events from 8 cells. We took total 2879 sEPSC events from control fast-spiking neurons: 37–645 events from 7 cells, and total 13,326 sEPSC events from LPS-exposed fast-spiking neurons: 191–2891 events from 9 cells.

[IPSC:] We took total 39,532 sIPSC events from control L5 pyramidal neurons: 1421–5643 events from 14 cells, and total 21,670 sIPSC events from LPS-exposed L5 pyramidal neurons: 578–4271 events from 10 cells, at least for 360 s. We took total 32,039 mIPSC events from control L5 pyramidal neurons: 376–5202 events from 11 cells, and total 28,582 mIPSC events from LPS-exposed L5 pyramidal neurons: 503–4455 events from 12 cells.

### 2.3 Statistical analysis

**Fig. 4:**
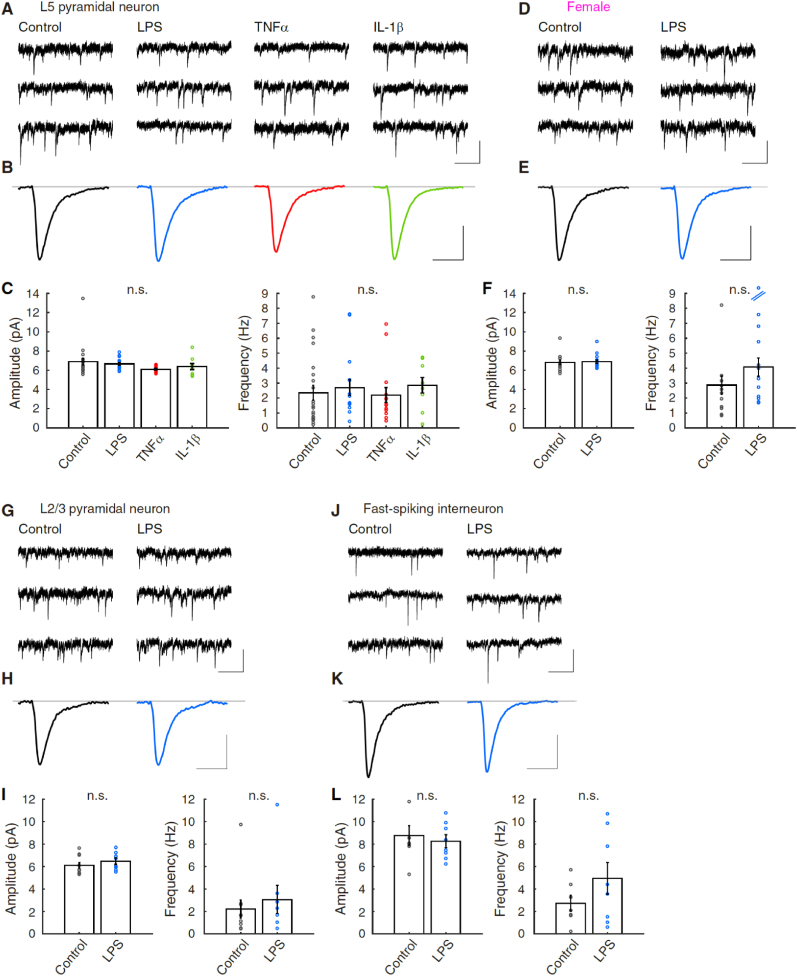
Transient activation of microglia does not affect spontaneous EPSC in the mPFC. **(A)** Representative traces of spontaneous excitatory postsynaptic currents (sEPSC) of the medial prefrontal cortex (mPFC) layer 5 (L5) pyramidal neurons of male rats in experiments of control, lipopolysaccharide (LPS), tumor necrosis factor α (TNF-α), and interleukin-1β (IL-1β). **(B)** Averaged representative sEPSC. 232, 171, 372, and 447 events were selected and averaged for the experiments of control, LPS, TNF-α, and IL-1β, respectively. **(C)** Bar graphs (mean ± SEM) of amplitude and frequency of sEPSCs in different experiments, respectively (control, n = 23; LPS, n = 16; TNF-α, n = 15; IL-1β, n = 9). No significant difference was gained by pairwise comparison at the significance level of 0.05 (n.s.). **(D)** Representative sEPSC traces of mPFC L5 pyramidal neurons of female rats in control and LPS-exposure. **(E)** Averaged representative sEPSC. 281 and 624 events were averaged for the experiments of control and LPS-exposure, respectively. **(F)** Bar graphs (mean ± SEM) of amplitude and frequency of sEPSCs in control and LPS experiments, respectively (control, n = 15; LPS, n = 16). No significant difference was observed for both amplitude and frequency by Mann-Whitney *U* test (n.s.). **(G)** Representative sEPSC traces of mPFC layer 2/3 (L2/3) pyramidal neurons of male rats in control and LPS-exposure. **(H)** Averaged representative sEPSC. 249 and 263 events were averaged for the experiments of control and LPS-exposure, respectively. **(I)** Bar graphs (mean ± SEM) of amplitude and frequency of sEPSCs in control and LPS experiments, respectively (control, n = 11; LPS, n = 8). No significant difference was observed by Mann-Whitney *U* test (n.s.). **(J)** Representative sEPSC traces of mPFC fast-spiking interneurons of male rats in control and LPS-exposure. **(K)** Averaged representative sEPSC. 136 and 342 events were averaged for the experiments of control and LPS-exposure, respectively. **(L)** Bar graphs (mean ± SEM) of amplitude and frequency of sEPSCs in control and LPS experiments, respectively (control, n = 7; LPS, n = 9). No significant difference was observed by Mann-Whitney *U* test (n.s.). Scale in (A), (D), (G), and (J): 10 pA, and 400 ms. Scale in (B), (E), (H), and (K): 4 pA, and 20 ms.

**Fig. 5:**
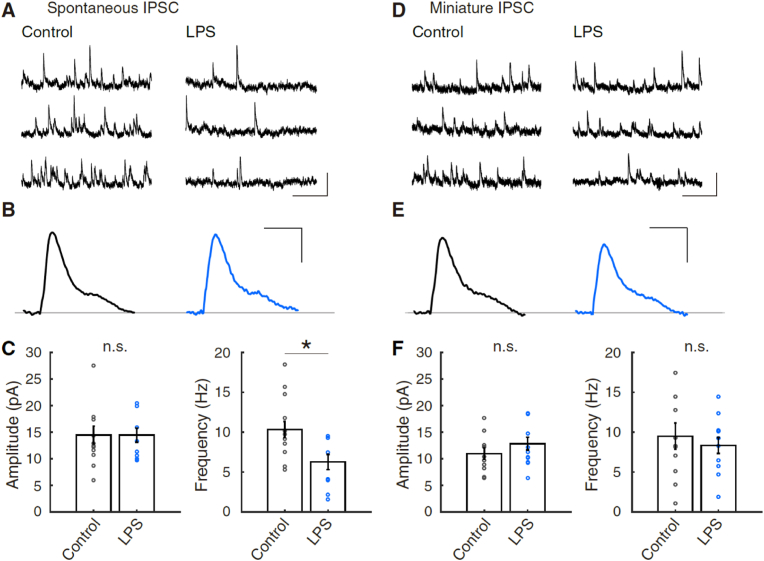
Reduction of the frequency of spontaneous IPSC of L5 pyramidal neurons by exposure to LPS. **(A)** Representative traces of spontaneous inhibitory postsynaptic current (sIPSC) of mPFC L5 pyramidal neurons in experiments of control and lipopolysaccharide (LPS)-exposure. **(B)** Averaged representative sIPSC. 366 and 180 events were selected and averaged for the experiments of control and LPS-exposure, respectively. **(C)** Bar graphs (mean ± SEM) of amplitude and frequency of sIPSCs in control and LPS experiments, respectively (control, n = 14; LPS, n = 10). An asterisk indicates a significant decrease between control and LPS-exposure (p < 0.02, ranksum = 82, with two-tailed Mann-Whitney *U* test). **(D)** Representative miniature IPSC (mIPSC) traces of mPFC L5 pyramidal neurons in control and LPS-exposure. **(E)** Averaged representative mIPSC. 162 and 166 events were averaged for the experiments of control and LPS-exposure, respectively. **(F)** Bar graphs (mean ± SEM) of amplitude and frequency of mIPSCs in control and LPS experiments, respectively (control, n = 11; LPS, n = 12). There are no significant differences (n.s.) among the pairs of amplitude and frequency (Mann-Whitney *U* test). Scale in (A) and (D): 20 pA, and 800 ms. Scale in (B) and (E): 4 pA, and 20 ms.

All the data are shown as the mean ± SEM across the group datasets unless otherwise stated. The complete results of the statistical analyses, including statistics, are reported in the Results section. Two-tailed Mann-Whitney *U*-tests were used to compare the data between two independent groups in Fig. 1C, **L, 4F,4I, 4L, 5C, and 5F**. In Fig. 2, Fig. 4C, we applied pairwise comparison between the subject (*p < 0.05). In the long-lasting recording of Fig. 1E, G, 1I, 1N, 1Q, 2B, 2F, 2H, 2J, 3A–3G, and S3B, we compared firing frequency normalized by 5-min average before 0 min (*i.e.*, −5 to −1 min) to that of 25–30 min later with a two-tailed Mann-Whitney *U* test at the significance level of p < 0.05. All the statistical analyses were performed using MATLAB R2015a and R2020a.

**Code/Software Accessibility.** Source data that support the findings of this study and custom MATLAB code for analyses are available from the corresponding author upon individual request.

## 3 Results

### 3.1 Distinct directionality of the endotoxin-induced plasticity of the intrinsic excitability in the cerebral cortices

To elucidate the neurophysiological modulations by immune activation, we examined the action potential firing as the neuronal excitability of mPFC pyramidal cells of layer 5 under slice preparation (Fig. 1A) following exposure to the endotoxin LPS, which activates microglia. First, we investigated the firing properties of L5 pyramidal neurons in the presence of picrotoxin to block fast GABAergic inhibitory transmission, by whole-cell patch-clamp recording and under current-clamp (Fig. 1B and C). After application of LPS (15 μg/mL) to neocortical slices in a bath-chamber, the firing frequency of neurons in response to depolarization step pulses with different amplitude of depolarization current (0–450 pA) was significantly lower than control (*p < 0.05 at more than 150 pA) (Fig. 1C).

To investigate the time course of firing-frequency changes, we subjected brain slices to transient LPS (15–20 μg/mL)-exposure in pyramidal neurons and continuously monitored subsequent firing properties for more than 30 min. The firing frequency following LPS-exposure showed a significant decrease relative to the baseline in mPFC L5 pyramidal neurons (*p < 0.05), while the control did not show any changes with significant difference (Fig. 1D and E). Therefore, in response to the LPS exposure, the mPFC pyramidal neurons decreased their firing frequency long-lastingly (Control: 14.9 ± 1.0 Hz at −5 to −1 min, and 15.4 ± 1.8 Hz at 25–30 min, 103.4 ± 10.4% of baseline, n = 7, p = 0.690, ranksum = 56, with two-tailed Mann-Whitney *U* test; LPS: 14.9 ± 1.7 Hz at −5 to −1 min, and 10.2 ± 1.4 Hz at 25–30 min, 68.8 ± 4.2% of baseline, n = 8, *p < 0.0005, ranksum = 100) (Fig. 1E). Otherwise, with low concentration of LPS (5–10 μg/mL), such modulation was not prominent (Figure S2). The direction of the modulation of firing frequency of L5 pyramidal neurons was distinct from that of the cerebellar Purkinje cells (Yamamoto et al., 2019). These results suggest the opposite directionality of the forms of plasticity of intrinsic excitability between both types of neurons.

We also tested the hypoexcitability plasticity under additional blockade of excitatory synaptic transmission (20 μM NBQX disodium salt and 50 μM APV). The firing frequency of the mPFC L5 pyramidal neurons following LPS-exposure showed a long-lasting decrease (11.3 ± 0.6 Hz at −5 to −1 min, and 7.4 ± 1.2 Hz at 25–30 min, 65.4 ± 8.7% of baseline, n = 6, *p < 0.003, ranksum = 57) (Fig. 1F and G), suggesting that decrease in the action potential is induced without both inhibitory and excitatory synaptic transmission. And we confirmed this endotoxin-triggered intrinsic plasticity in the preparation of female animals to a similar extent (11.0 ± 0.5 Hz at −5 to −1 min, and 7.4 ± 1.0 Hz at 25–30 min, 66.4 ± 7.9% of baseline, n = 8, *p < 0.0002, ranksum = 100) (Fig. 1H and I), suggesting without gender bias. Although we measured the waveform of action potentials of L5 pyramidal neurons to test changes in the parameters of action potentials between with and without exposure to LPS, there were no significant differences in any parameters of action potential waveform (: basement voltage, action potential latency, peak, threshold, amplitude to the peak, full width at half maximum, 10–90% rise time, afterhyperpolarization) (Control, n = 7; LPS-exposure, n = 8, with pairwise comparison; *data not shown*). The result implies that acute stimulation of microglia does not alter the peak and threshold of action potentials, reflecting the activity of active ion channels such as voltage-sensitive Na^+^ channels. It is notable that chronic application of TTX and suppression of the activity of neurons downregulate the Na^+^ and leak K^+^ channels (Desai et al., 1999).

Further, we asked if the firing frequency changes by injecting test depolarization pulses with 20-sec intervals. To clarify whether firing frequency changes when applying a limited number of current pulses before and after LPS, we administered the test depolarization pulses at the rate of every 60 s (*i.e.*, every 1 min) (Figure S3). We confirmed the decrease in the firing frequency of evoked action potentials after transient LPS-exposure (56.0 ± 7.1% at 25–30 min, Figure S3) to the extent of Fig. 1D–I, as well as no substantial changes in the firing frequency at every 1-min depolarization at least for 10 min in test period (Figures S3C & S3D). This result suggests that the combination of the LPS-exposure and depolarization (: associated with evoked action potentials at the interval) is not required for the induction of hypoexcitability plasticity, but sole LPS-exposure is sufficient. Our results in Fig. 1B & C also support the scenario because firing frequency was decreased after LPS-exposure while recorded from the neurons without applying any depolarization through pipettes. Nevertheless, we have not applied test depolarization at a higher frequency vice versa. Provided that, the firing frequency might change in the opposite direction (*i.e.*, intrinsic plasticity or hyperexcitability), since depolarizing current at 10 Hz to L2/3 pyramidal cells increased the action potential firing long-lastingly (Gill and Hansel, 2020), while it is beyond our scope and an open question.

Next, we tested if LPS-exposure modulates the intrinsic excitability of L2/3 pyramidal neurons and fast-spiking interneurons (Fig. 1J–Q). The firing frequency of L2/3 pyramidal neurons of control and after application of LPS indicates that L2/3 pyramidal neurons also show a significant reduction of the excitability against LPS-exposure (*p < 0.05 at more than 300 pA pulses) (Fig. 1J–L). To investigate the time course of firing-frequency changes, we subjected brain slices to transient LPS-exposure in L2/3 pyramidal neurons. The firing frequency following LPS-exposure also showed a significant decrease relative to the baseline in mPFC L2/3 pyramidal neurons long-lastingly (17.8 ± 2.1 Hz at −5 to −1 min, and 11.9 ± 2.5 Hz at 25–30 min, 64.9 ± 9.0% of baseline, n = 6, *p < 0.003, ranksum = 57) (Figures 1M and N). In contrast to both pyramidal neurons, the time course of firing-frequency changes of fast-spiking interneurons following LPS-exposure showed no significant changes relative to the baseline (24.8 ± 4.7 Hz at −5 to −1 min, and 29.4 ± 6.9 Hz at 25–30 min, 114.3 ± 11.9% of baseline, n = 6, p = 0.3636, ranksum = 33) (Fig. 1O–Q), suggesting different modulation mechanisms among cell-types in response to LPS and activated microglia in the mPFC. While we don't show the relationship between injection current and firing frequency, the rheobase of fast-spiking neurons is small (: 50–100 pA) and the basal firing frequency is high (: 11.9–45.3 Hz) (Figures S1E and S1F).

### 3.2 Involvement of SK channels for the decrease in the intrinsic excitability of mPFC pyramidal neurons

Previously, Yamamoto et al. (2019) demonstrated an increase in the firing frequency of cerebellar Purkinje cells via microglia activation by exposure to LPS. Activated microglia mainly secrete the inflammatory cytokine, TNF-α. Increased intrinsic excitability results from the downregulation of SK-channel function, which is considered to share the signal necessary for the induction of IP of Purkinje cells (Schonewille et al., 2010; Belmeguenai et al., 2010; Ohtsuki et al., 2012; Grasselli et al., 2016, 2020; Ohtsuki and Hansel, 2018; Ohtsuki, 2020). In contrast, we observed a decrease in the firing frequency of pyramidal cells after exposure to LPS in the mPFC. We speculated a possibility of the mechanism involving modulation of SK-channel functions, and we focused on the modulation of L5 pyramidal neurons in the following experiments of this study. Then we applied an agonist of SK channels, CyPPA (10 μM) continuously, and found that the application of CyPPA decreased the firing frequency of L5 pyramidal neurons (Fig. 2A and B). After application of CyPPA, the firing frequency of neurons decreased from 16.5 ± 0.9 Hz to 8.1 ± 1.3 Hz (n = 8, *p < 0.0005, ranksum = 100) (Fig. 2C). The extent of decrease was 49.1 ± 7.2% of the baseline (n = 8, *p < 0.001) (Fig. 2B and D). Compared to the extent of decrease in firing frequency by exposure to LPS, the decrease in firing frequency by CyPPA appeared to be saturated. SK channels are subdivided into SK1 (KCa2.1), SK2 (KCa2.2), and SK3 (KCa2.3). CyPPA is an activator of SK channels with selectivity for SK2 and SK3 channels but with no activity at SK1 and KCa3.1 (IK) channels. Due to the specificity of CyPPA against the SK-channel subfamily, the decrease in action potential firing by application of CyPPA suggests the expression of SK2 and SK3 subtypes in the mPFC pyramidal cells. Next, we applied CyPPA to pyramidal neurons that were exposed to LPS beforehand. Then, the firing frequency of neurons did not decrease anymore (CyPPA after LPS: from 13.8 ± 1.9 Hz to 13.1 ± 1.9 Hz, 96.0 ± 8.3% at 25–30 min, n = 6, p = 0.136, ranksum = 48) (Fig. 2A–D, CyPPA after LPS). The impairment of the firing reduction by CyPPA after LPS-exposure suggests that SK2 and SK3 are irrelevant to the hypoexcitability of pyramidal neurons.

In the cerebellar Purkinje cells, less than 10-nM order of apamin, an SK channel antagonist, sufficiently increases the intrinsic excitability (Belmeguenai et al., 2010; Ohtsuki et al., 2012; Ohtsuki, 2020), and modulation of the SK2 subtype function is crucial for the intrinsic plasticity (Grasselli et al., 2020). Currently, the effect of apamin on the intrinsic excitability in the cerebral cortex is quite controversial. In hippocampal pyramidal neurons, blockade of SK channels by 100 nM apamin is reported not effective for modulating the firing frequency (Gu et al., 2008; Chen et al., 2014), although a recent study indicated the increase in the firing frequency of the L5 pyramidal neurons in the somatosensory cortex by application of 100 nM apamin (Bock et al., 2019).

In this study of the mPFC L5 pyramidal neurons, application of 100 nM (n = 2, *data not shown*) and 500 nM (n = 4) apamin to the mPFC slice did not change the firing frequency of L5 pyramidal neurons in our experimental condition (10.7 ± 0.6 Hz to 10.5 ± 1.5 Hz; 96.5 ± 10.2%, n = 4, p = 0.753 with two-tailed Mann-Whitney *U* test) (Fig. 2E and F). To investigate the involvement of SK channels for the immune-triggered intrinsic plasticity, we applied 100 nM apamin to the mPFC slice exposed to LPS (20 μg/mL). Given SK channels are functionally upregulated via LPS-exposure, the following blockade of the high-affinity SK channels against apamin should increase the firing frequency. However, 100 nM apamin did not alter the firing frequency of L5 pyramidal neurons (8.2 ± 1.6 Hz to 8.3 ± 1.9 Hz; 105.5 ± 17.1%, n = 5, p = 0.683 with two-tailed Mann-Whitney *U* test) (Figs. 2H and 100 nM). Meanwhile, application of 500 nM apamin to the mPFC slice pre-exposed to LPS increased the firing frequency of L5 pyramidal neurons (7.2 ± 2.3 Hz to 10.2 ± 0.8 Hz; 146.0 ± 9.2%, n = 7, *p < 0.0006 with two-tailed Mann-Whitney *U* test) (Figs. 2H and 500 nM). According to Stocker et al. (2004), IC50 of apamin against SK1, SK2, and SK3 channels are 0.70 – >100 nM, 0.027–0.14 nM, and from 0.63 to 25 nM, respectively (Stocker et al., 2004; Stocker, 2004). Cerebellar Purkinje cells dominantly express SK2, and pyramidal neurons in the forebrain express SK1–SK3 proteins (Sailer et al., 2002; Stocker, 2004; Adelman et al., 2012; Adelman, 2016). Considering low affinity for apamin of SK1 and SK3, those subtypes are suggested to be involved in the hypoexcitability plasticity of mPFC L5 pyramidal neurons, other than SK2.

Considering the results of CyPPA and apamin experiments, substantially no effects of CyPPA after LPS-exposure (Fig. 2B) suggest that the SK3 is not activated anymore after LPS-exposure. Then, SK1 is suggested to be upregulated by LPS exposure and microglia activation in mPFC. This interpretation is in contrast to the downregulation of SK2 channels in the cerebellar Purkinje cells (Yamamoto et al., 2019). Together, these results suggest that the upregulation of SK channel function underlies the decrease in intrinsic excitability of pyramidal neurons. In this study, we used the word “upregulation” to mean an increase of ion-channel function in distinct ways: the channel molecule translocates in the cellular space and cellular membrane by a certain exocytosis mechanism, or the channel molecule changes its conductance by a certain modulatory mechanism.

Lastly, to investigate the involvement of BK channels (KCa1.1) for the immune-triggered intrinsic plasticity, we applied 200 nM charybdotoxin to the mPFC slice exposed to LPS. But 200 nM charybdotoxin did not change the firing frequency of the pyramidal neurons (6.6 ± 0.7 Hz to 6.3 ± 1.3 Hz; 91.1 ± 11.9%, n = 6, p = 0.136 with two-tailed Mann-Whitney *U* test), suggesting the irrelevance of the BK channels to immune-triggered plasticity (Fig. 2I and J). Charybdotoxin does not only block BK channels but also voltage-dependent K^+^ channels (Kv1.2 and Kv1.3). Thus, the results imply those channels are not involved, either.

### 3.3 Involvement of microglial activity and inflammatory cytokine signal for the decrease in the intrinsic excitability of mPFC pyramidal neurons

After exposure to LPS, microglia are considered to release neuromodulatory substances. To examine if plasticity of firing frequency is induced under inhibition of microglia activity, we applied LPS to the brain slices incubated with PLX3397, an inhibitor of the colony-stimulating factor 1 receptor (CSF-1R) kinase (Ohno et al., 2006; Elmore et al., 2014). The CSF-1R expresses in all microglia and their signaling is required for microglial maintenance. It is also known that neocortical pyramidal neurons do not express CSF-1Rs (Chitu et al., 2016). Deletion or long-term blockade of CSF-1R causes widespread microglial depletion (Ginhoux et al., 2010; Nandi et al., 2012; Elmore et al., 2014; Yamamoto et al., 2019). As we expected, the decrease in the firing frequency of mPFC pyramidal neurons by exposure to LPS was prevented by PLX3397 (10 μM)-preincubation for 1 h (LPS + PLX3387 to L5 pyramidal neurons: from 11.7 ± 0.7 Hz to 11.8 ± 0.8 Hz, 100.6 ± 3.8% at 25–30 min, n = 7, p = 1, ranksum = 52.5), suggesting that microglia activity mediates the induction of the hypoexcitability plasticity in the mPFC pyramidal neurons (Fig. 3A). Thus, the inhibition of CSF-1R kinase of microglia is suggested to suppress the induction of the LPS-triggered decrease in the firing frequency of mPFC pyramidal cells. In sum, the result suggests that the blockade of CSF-1R signal and suppression of microglial function prevented the induction of immune-triggered changes of intrinsic excitability in L5 pyramidal neurons, and thus, mPFC immune cell also changes neuronal activity.

Activated microglia release TNF-α and interleukin (IL)-1β in the early phase of their activation. Next, we investigated if the decrease in the intrinsic excitability of L5 pyramidal neurons is mediated by the release of TNF-α or IL-1β. We first applied 100–150 ng/mL of TNF-α via perfusion, and it decreased the frequency of the action potential firing (: from 11.7 ± 1.1 Hz to 6.9 ± 0.7 Hz, 60.6 ± 6.0% at 25–30 min, n = 7, *p < 0.0006, ranksum = 77) (Fig. 3B). We noticed a relatively higher concentration of TNF-α was effective, compared to the cerebellar Purkinje cells (Yamamoto et al., 2019). An inhibitor of TNF-α, C 87 (40 μM) together with LPS (15–20 μg/mL) in the bath chamber did not change the firing frequency after the application of reagents (LPS + C 87 to L5 pyramidal neurons: from 10.5 ± 0.8 Hz to 10.9 ± 1.1 Hz, 103.1 ± 3.7% at 25–30 min, n = 6, p = 0.682, ranksum = 36) (Fig. 3C). In contrast, application of 3 nM IL-1β did not change the frequency significantly (: from 12.6 ± 1.5 Hz to 12.2 ± 1.7 Hz, 96.0 ± 6.2% at 25–30 min, n = 7, p = 0.3636, ranksum = 59.5) (Fig. 3D). These data suggest that the decrease in firing frequency by exposure to LPS was mediated by TNF-α but not by IL-1β.

### 3.4 Intracellular signaling for the decrease in the intrinsic excitability of mPFC pyramidal neurons

**Fig. 6:**
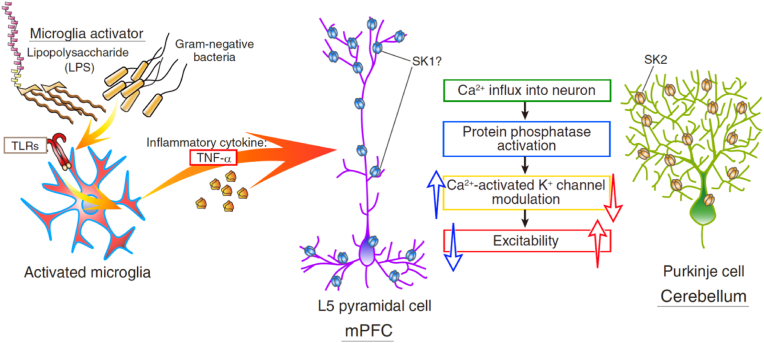
Comparison of endotoxin-microglia-TNF-α signaling of forms of the plasticity of intrinsic excitability of mPFC L5 pyramidal cells and cerebellar Purkinje cells. Exposure to LPS, bacterial endotoxin, activates microglia, in both the mPFC and the cerebellum. Activated microglia release inflammatory cytokines including TNF-α, which modulates the SK channel functions in both pyramidal neurons and Purkinje cells. The directionality of the immune-triggered plasticity of intrinsic excitability is different between mPFC L5 pyramidal neurons and cerebellar Purkinje cells, suggesting the distinct mechanisms of modulation of SK channels among neuronal types or functional heterogeneity of microglia.

In the case of cerebellar Purkinje cells, it is already revealed that the secreted TNF-α modulates SK-channel function through the activation of downward signaling of TNF-receptors and the activation of intraneuronal PP2B (Pribiag and Stellwagen, 2013; Yamamoto et al., 2019). First, to investigate the Ca^2+^-dependence for the LPS-triggered decrease in the intrinsic excitability of mPFC neurons, we applied the Ca^2+^ chelator BAPTA into the pipette-solution and monitored the firing frequency of neurons continuously. The aim of this experiment is not to examine the effect of the Ca^2+^ chelator against the action potential generation in the pyramidal neurons but to see the effect for the plasticity induction. The range of firing frequency of mPFC L5 pyramidal neurons filled with BAPTA was 10.9 Hz–19.9 Hz (Figure S1F), and the exposure to LPS did not change the firing frequency of mPFC pyramidal neurons (LPS to L5 pyramidal neurons fulfilled BAPTA: from 15.4 ± 1.3 Hz to 15.6 ± 0.9 Hz, 100.6 ± 3.0% at 25–30 min, n = 7, p = 1, ranksum = 52.5) (Fig. 3E). Generally, calcium chelators, *e.g.*, BAPTA, should work as a blocker of SK channels immediately after rupture of the membrane. The impairment of LPS-triggered plasticity under a very low concentration of Ca^2+^ suggests that intracellular Ca^2+^ is required for induction of the plasticity, although SK-channel-activity would be already blocked. Therefore, the decrease in the firing frequency of L5 pyramidal neurons is suggested to be Ca^2+^-dependent. Next, to examine the intracellular mechanism of upregulation of SK channels in the mPFC pyramidal neurons, we pharmacologically inhibited the activity of broad protein phosphatases by filling a pipette solution that includes okadaic acid (300 nM), a broad inhibitor of protein phosphatases 1, 2A, and 2B. We found that the microglia-triggered decrease in the intrinsic excitability was abolished by the intra-neuronal blockade of phosphatases (LPS to L5 pyramidal neurons fulfilled okadaic acid: from 13.8 ± 0.8 Hz to 13.5 ± 1.0 Hz, 97.8 ± 2.9% at 25–30 min, n = 6, p = 0.364, ranksum = 45) (Fig. 3F). We lastly applied cyclosporin A, an inhibitor of PP2B (also called calcineurin, a calcium/calmodulin-activated serine/threonine phosphatase) and found that intraneuronal cyclosporin A prevented the induction of LPS-triggered decrease in the firing frequency (LPS to L5 pyramidal neurons fulfilled cyclosporin A: from 12.4 ± 0.9 Hz to 12.1 ± 1.4 Hz, 96.4 ± 5.5% at 25–30 min, n = 7, p = 0.180, ranksum = 63) (Fig. 3G). In Figure S1, we show the basal membrane potential and those input resistances throughout the experiments, which indicate substantial stability of the experiments. The current-clamped input resistance is the sum of membrane resistance and series resistance. In recordings, the membrane resistance did not change much, though our results suggest an increase in SK-channel function. It is because we applied hyperpolarizing-current to gain the input resistance, whereas depolarization pulses should open low voltage-activated calcium channels and they would activate SK channels. Depolarization test pulses do not affect the input resistance. Hyperpolarization-activated K + -channels (*i.e.,* HCN channels) could potentially contribute to our input resistance. However, there is no change in the input resistance of pyramidal cells (as shown in Figure S1), implying less involvement of their modulation after LPS-exposure. Thus, these results suggest that, in the early phase of LPS-exposure, mPFC L5 pyramidal neurons decrease their firing frequency via TNF-α-release from microglia, activation of intraneuronal PP2B, and upregulation of presumably SK1 function. Compared to the increase in the firing rate in cerebellar Purkinje cells, the directionality of the immune-triggered plasticity is inversed in L5 pyramidal cells in the mPFC (Fig. 6).

### 3.5 Synaptic transmissions in response to endotoxin-exposure in mPFC neurons

We next monitored the spontaneous and miniature events of excitatory and inhibitory synaptic transmission (: sEPSC, sIPSC, and mIPSC) of L5 pyramidal neurons before and after exposure to LPS. The amplitude and frequency of sEPSC of cerebellar Purkinje cells were both increased by LPS-exposure (Yamamoto et al., 2019). In contrast, the amplitude and frequency of sEPSC of L5 pyramidal neurons were neither significantly changed in the mPFC by LPS-exposure (sEPSC amplitude, Control: 6.9 ± 0.3 pA, n = 23; LPS: 6.6 ± 0.2 pA, n = 14: sEPSC frequency, Control: 2.3 ± 0.5 Hz, n = 23; LPS: 2.5 ± 0.5 Hz, n = 14), nor by exposure to TNF-α and IL-1β (sEPSC amplitude, TNF-α: 6.1 ± 0.1 pA, n = 23; IL-1β: 6.5 ± 0.3 pA, n = 14: sEPSC frequency, TNF-α: 2.2 ± 0.5 Hz, n = 23; IL-1β: 2.8 ± 0.5 Hz, n = 14) (p = 0.0905 in sEPSC amplitude, and p = 0.298 in sEPSC frequency by multiple comparisons using rank sums) (Fig. 4A–C). sEPSC recorded from female rats showed no differences in both amplitude and frequency (p = 0.767, ranksum = 232 in amplitude, and p = 0.0927, ranksum = 197 in frequency) (Fig. 4D–F). Thus, in L5 pyramidal neurons, it is suggested that exposure to LPS does not change the excitatory synaptic transmission significantly at the recording from somata, although the presynaptic excitatory neurons may change their excitability.

Further, we recorded sEPSC from the L2/3 pyramidal neurons and fast-spiking interneurons, and we found no significant differences between before and after LPS-exposure in their amplitude and frequency in both neurons (L2/3 pyramidal neurons: p = 0.300, ranksum = 93 in amplitude, and p = 0.430, ranksum = 90 in frequency, Fig. 4G–I; fast-spiking interneurons: p = 0.918, ranksum = 61 in amplitude, and p = 0.299, ranksum = 49 in frequency, Fig. 4J–L).

Lastly, we recorded sIPSC from L5 pyramidal neurons and found the reduction of the sIPSC frequency but not of the amplitude (sIPSC amplitude, Control: 14.4 ± 1.7 pA, n = 14; LPS: 14.4 ± 1.4 pA, n = 10: sIPSC frequency, Control: 10.3 ± 1.0 Hz, n = 14; LPS: 6.2 ± 1.0 Hz, n = 10) (p = 0.838, ranksum = 129 in sIPSC amplitude, and *p = 0.0127, ranksum = 82 in sIPSC frequency) (Fig. 5A–C). The activity of presynaptic neurons reflects spontaneous synaptic events. To eliminate its contribution, we measured the mIPSC from L5 pyramidal neurons in the presence of TTX. However, there were no significant differences between before and after LPS-exposure in their amplitude and frequency (mIPSC amplitude, Control: 10.9 ± 1.1 pA, n = 11; LPS: 12.8 ± 1.2 pA, n = 12: mIPSC frequency, Control: 9.5 ± 1.6 Hz, n = 11; LPS: 8.3 ± 1.0 Hz, n = 12) (p = 0.372, ranksum = 117 in sIPSC amplitude, and p = 0.689, ranksum = 139 in sIPSC frequency) (Fig. 5D–F), suggesting that the presynaptic release probability and postsynaptic responsiveness of GABAergic synaptic transmission in the mPFC L5 pyramidal neurons were not changed by LPS-exposure. Rather, the inhibitory synaptic transmission and the network activity in the mPFC were modulated after reducing the intrinsic excitability via microglia activation.

## 4 Discussion

In the early phase of microglia activation by exposure to LPS, the intrinsic excitability of neurons is modulated in different ways. In the cerebellar cortex, the excitability of Purkinje cells increases via downregulation of SK channels (Fig. 6) (Yamamoto et al., 2019), whereas the excitability of mPFC pyramidal neurons decreases via upregulation of SK channels in response to microglia activation (Figs. 1, Figure 2, and Fig. 3) and the release of inflammatory cytokine TNF-α (Fig. 3B and C). TNF-α is known to be secreted in the early phase of microglia activation, and the receptors expressing on the neuronal membrane convey the signaling to the intracellular cascade, including the activation of protein phosphatases (Fig. 3E–G) (Beattie et al., 2002; Santello et al., 2011; Pribiag and Stellwagen, 2013; Habbas et al., 2015; Yamamoto et al., 2019). We compared the modulation of neurophysiological properties representing the firing frequency of both cerebellar Purkinje cells and mPFC pyramidal cells. And we found the inverted directionality of the excitability modulation between both types of neurons (Fig. 6). Inverted directionality of immune-triggered intrinsic excitability plasticity would be derived from both neuronal and microglial mechanisms. In the following sections, we would like to discuss it.

### 4.1 Inverted directionality of immune-triggered plasticity

Interestingly, the endotoxin-triggered modulation of the intrinsic excitability gave an opposite consequence: hyper- and hypo-excitability in the cerebellum and mPFC, respectively. This relationship reminds us of the inverted directionality of synaptic plasticity between the cerebellar Purkinje neurons and the neocortical pyramidal neurons (Jörntell and Hansel, 2006). In both our results of the plasticity of intrinsic excitability, the experiments using agonist and antagonist suggested hyper- and hypo-excitability were caused by down- and up-regulation of SK channels in two types of neurons (Yamamoto et al., 2019, and Fig. 2 in this study). Therefore, it may imply that the intraneuronal modulation of the SK-channel function is inverted among both types of neurons. While many studies in the field of neuroinflammation suggested the increases in the inflammatory cytokines in samples of blood and brain parenchyma (Elmore et al., 2014; Masi et al., 2015; Matta et al., 2019; Yamauchi et al., 2021), those studies have not allowed us to interpret either the changes of the neuronal excitability or their differences among brain regions. Several ion-channels are involved in modulation of the intrinsic excitability of neurons via immune interaction (*e.g.*, SK channel, KV7/M channel, Cl-transporter, and presumably voltage-activated Na^+^ channels) (Coull et al., 2005; Pascual et al., 2012; Klapal et al., 2016; Tzour et al., 2017; Yamamoto et al., 2019). To our knowledge, the SK channel is the first ion channel in which the inverted modulation between the cerebellum and the neocortex was shown. As aforementioned, Jörntell and Hansel (2006) pointed out the inverted threshold of the induction of synaptic plasticities, such as LTP and LTD, comparing between the cerebellar Purkinje cells and neocortical pyramidal cells. For the induction of synaptic LTD in the Purkinje cells, a higher concentration of intracellular Ca^2+^ is required, while the synaptic LTP of pyramidal neurons demands the larger Ca^2+^ influx into the neurons than that for the induction of LTD (Wang et al., 2000; Wang et al., 2003; Jörntell and Hansel, 2006; Tanaka et al., 2007; Ohtsuki et al., 2009). Activation of Ca^2+^-dependent protein phosphatase (*i.e.*, PP2B) is involved in the induction of synaptic LTP in Purkinje cells and LTD in the pyramidal neurons. Signaling after the protein phosphatase is thought to promote the trafficking of AMPA receptors into and out of the postsynaptic membrane of Purkinje cells and pyramidal neurons, respectively (Ehlers, 2000; Chetkovich et al., 2002; Coesmans et al., 2004; Jörntell and Hansel, 2006). Since the trafficking mechanism of SK-channel molecules is not fully understood yet (Adelman, 2016), we can only speculate that the mechanism among the neurons would be different, and probably the trafficking of SK channels might share or relate to that of glutamate-receptor subunits. While we intended to compare the immune-triggered plasticity induction in different brain regions, it is inevitable to have a difference in the experimental condition among studies for the in vitro recordings. It is due to the distinction of the ideal recording conditions for each brain region and cell type. According to Gill and Hansel (2020), L2/3 pyramidal neurons in the primary somatosensory cortex (S1) also exhibit intrinsic plasticity accompanied by an increase in excitability (Gill and Hansel, 2020). The authors used SK2KO mice and showed downregulation of the SK2 channel by protein kinase A (PKA) and casein kinase 2 (CK2) (Gill and Hansel, 2020). On the other hand, upregulation of the SK channel (, presumably SK1) was suggested involved in the decrease in the intrinsic excitability of mPFC pyramidal neurons from results in this study (Fig. 2). While the induction of the hypoexcitability plasticity would share the mechanism between L2/3 and L5 pyramidal neurons, the precise mechanism remains unrevealed from our experiments (Figures 1M and N). Inhibitory fast-spiking interneurons did not express a form of intrinsic plasticity but showed a slender increase in the firing frequency (Figures 1P and Q). Their molecular mechanism for modulation should be distinct from the pyramidal neurons. Therefore, the directionality of modulation of the SK-channel function during plasticity is thought dependent on phosphatase and kinase activity, and cell types.

### 4.2 Heterogeneity of the microglia against the region-dependence of endotoxin-triggered plasticity

Compared to the previous study in the cerebellar Purkinje cell (Yamamoto et al., 2019), a higher concentration (15–20 μg/mL) of LPS was required for the induction of the hypoexcitability plasticity in the pyramidal neurons (Fig. 1 and Figure S2), and relatively higher concentration (100–150 ng/mL) of TNF-α was effective for the reduction of firing frequency (Fig. 3B). From those results, the reactivity of microglia may not be identical between the mPFC and the cerebellum. The fact that all the microglia are revealed to originate from the same early yolk sac progenitors (Ginhoux et al., 2010; Kierdorf et al., 2013; Matcovitch-Natan et al., 2016) may have led ones to believe that microglia are homogeneous in their phenotypes and functions. Early progenitors of microglia infiltrate the brain parenchyma and reside there before the blood-brain barrier is functionally completed. They are considered to become the first kernels for innate immune in the CNS. Because of this nature, microglia are often considered homogenous throughout the brain. However, currently, it is known that microglia are not so homogenous, but with different functions, structures, and cellular profiles across brain regions (Stoessel and Majewska, 2021), and the relevance to the neurophysiological functions is less understood. How microglia obtain different features in different milieus may be explained by regional specifications, even including an effect of the vasculature system, secreted cytokines, and growth factors (Segawa et al., 2021). Though it is beyond our scope, several studies indicate that microglia are heterogeneous across brain regions in terms of morphology, signaling pathways, and gene expression patterns (Ayata et al., 2018; Grabert et al., 2016; Silvin and Ginhoux, 2018; Süß et al., 2020; Stoessel and Majewska, 2021). For instance, in the basal ganglia, such as the ventral tegmental area (VTA) and the substantia nigra, microglia have distinct morphologies and transcriptional profiles (De Biase et al., 2017). Microglia in the cerebellum are less ramified and are sparsely distributed within the tissue (Ashwell, 1990; Lawson et al., 1990; Vela et al., 1995; Stowell et al., 2018). Interestingly, they appear to stretch the processes differently in horizontal and sagittal directions. While cortical microglia have been intensively studied, they would not be representing all the microglial populations in the brain. Therefore, it is important to study the effect of microglial activation in different brain regions, and our results may indicate such different functions at the aspect of neurophysiology. It would be interesting to investigate if long-lasting and chronic manipulation of microglia activity may change the intrinsic excitability via modulation of voltage-sensitive Na^+^ channels and leak K^+^ channels like homeostatic plasticity (Desai et al., 1999; Turrigiano et al., 1994), although it is also beyond our scope. Further experiments should answer the question.

### 4.3 Acute immune activation modifies synaptic transmission and intrinsic excitability of neurons

The proliferation of activated immune cells in the parenchyma disrupts various physiological properties of neurons there (Davalos et al., 2012; Nie et al., 2018). Consequently, they could cause a hyper- and hypo-excitability of the intrinsic membrane properties decided by the function of K^+^, Na^+^, Ca^2+^ channels, and other transporters (Yamamoto et al., 2019; Ozaki et al., 2021; Segawa et al., 2021). Microglia-mediated synaptic and structural plasticity depends upon a number of signaling pathways including complement signaling (Stevens et al., 2007; Schafer et al., 2012), purinergic signaling (Sipe et al., 2016), adrenergic signaling (Stowell et al., 2019), fractalkine signaling (Hoshiko et al., 2012), neurotrophic factors (Parkhurst et al., 2013), and superoxide (Zhang et al., 2014), other than the scenario of proinflammatory cytokines. However, our result of acute inflammation did not indicate any changes in sEPSC of L5 and L2/3 pyramidal cells, and fast-spiking interneurons by recordings from somata (Fig. 4). Further, the previous interpretation of several studies is compromised in that changes in synaptic transmission are arbitrarily directed to the changes in intrinsic excitability (*e.g.*, firing frequency). An increase and a decrease in spontaneous or miniature EPSC do not always indicate the changes in the intrinsic excitability of every neuron. Or, the emerged phenomena would be just an aspect at the timing of observation. In this study, we found a decrease in the spontaneous IPSC frequency but no changes in mIPSC amplitude and frequency (Fig. 5). Considering the basal frequency of sIPSC (∼10.3 Hz) and sEPSC (∼2.3 Hz) of L5 pyramidal neurons, the substantial impact for the network activity would be higher in the inhibitory synaptic transmission, while almost 10–20% of cells are inhibitory interneurons in the neocortex. The decreased excitability of the pyramidal neurons via microglia activation may decrease the net activity in the mPFC and may result in the reduction of sIPSC frequency.

### 4.4 The relevance of neuroinflammation to psychiatric diseases

Accumulating evidence prevailed a notion that microglia are associated with not only inflammation, but also emotional or mood-related psychiatric diseases accompanied by psychological stress or morbidity. While the full physiological effects of immune-related responses in CNS remain unclear (Paolicelli et al., 2011; Aguzzi et al., 2013; Parkhurst et al., 2013; Xanthos and Sandkühler, 2014; Chung et al., 2015; Tay et al., 2017; Yamamoto et al., 2019; Ozaki et al., 2021), it is known that TNF-α is released during the activation of microglia in the early phase of microbial and viral infections, and the high concentration of TNF-α persists for a while. In human ASD patients, the TNF-α level of M1/M2 macrophage is increased (Yamauchi et al., 2021). According to Onore et al. (2013) in a mouse model of ASD, an elevation of the inflammatory cytokine levels (: IL-6, MCP-1 (as CCL2), macrophage inflammatory protein (MIP)-1α (as CCL3), IL-12 (p40), IL-12 (p70), IL-6, and TNF-α) is linked to the extent of the autistic-like behavior (Onore et al., 2013). Thus, an increased level of the brain inflammatory cytokines and the modulation of neuronal activity in the wide brain regions would be associated with the dysfunction of brain activity and the emergence of psychiatric disorders. In fact, the cerebello-frontal pathways may be susceptible to the disease models. The cerebellum is now thought one of the distinct regions that cause the emergence of motor-discoordination, ASD, and depression-like behaviors (Shih et al., 2015; Mitoma et al., 2016; Stoodley et al., 2017; Yamamoto et al., 2019; Ohtsuki et al., 2020). Prenatal exposure to infection, like sepsis of pregnant mothers, results in modulation of synaptic protein expression in the hippocampus, neocortex, frontal cortex, and cerebellum, accompanied by motor impairment in neonates, depressive-like behavior, memory, and learning impairments (Granja et al., 2021). Recently, periaqueductal gray (PAG) and VTA have been revealed to be involved in such behaviors (Vaaga et al., 2020; Carta et al., 2019). The glutamatergic neurons in the cerebellar nuclei are shown to project directly to the PAG through the cerebellar aqueduct, whereas they are also implemented to the functional circuit of cerebellar-VTA (Vaaga et al., 2020; Carta et al., 2019). In turn, the PAG is involved in the autonomous nerve and emotional behaviors, and the VTA regulates the dopamine release to the mPFC. Additionally, inflammation in the prefrontal cortex causes depression-like behaviors and anxiety in mice (Nie et al., 2018; Zheng et al., 2021). Perhaps, in the animals with infections of inflammatory microorganisms and viruses, the inflammation may disrupt the functional connectivity of brain activity due to the hyper-, and hypo-activity of neurons through the immune-triggered plasticity of the intrinsic excitability in various brain regions. While it has begun to be understood the circuit projecting from and to the prefrontal cortex impacting the whole brain network (Kelly and Strick, 2003; Suzuki et al., 2012), the cerebello-prefrontal pathways and the immune-triggered intrinsic plasticity could be involved in the psychiatric diseases (Yamamoto et al., 2019; Kelly et al., 2020; Ohtsuki et al., 2020; Ozaki et al., 2021).

## 5 Conclusions

Our study shows that the directionality of the microglia-triggered intrinsic plasticity is inverted between the mPFC L5 pyramidal cells and the cerebellar Purkinje cells. The induction of the hypoexcitability plasticity of mPFC L5 pyramidal neurons is mediated by the TNF- α and is dependent on the intraneuronal activity of protein phosphatases. The functional upregulation of SK1 channels is involved in the LTD of mPFC pyramidal cells. The contrasting responses against acute inflammation in the two brain regions may cause the impairment of network functional connectivity during immune-associated brain dysfunctions.

## Animal ethics statement

All procedures were performed following the guidelines of the Animal Care and Use Committees and approved by the Ethical Committee of Kyoto University. All animal handling and reporting comply with the ARRIVE guidelines. Rats were housed (5 animals at maximum in each cage) and maintained under a 12-h light: 12-h dark cycle, at a constant temperature and humidity (20–24 °C, 35%–55%), with food and water available ad libitum.

## Funding sources

This work was supported by grants from the 10.13039/100012131Brain Science Foundation, the 10.13039/100011313Tokyo Biochemical Research Foundation, the 10.13039/100007428Naito Foundation, the 10.13039/501100004398Mitsubishi Foundation, the 10.13039/100007449Takeda Science Foundation, and the Hakubi-project grant (10.13039/501100005683Kyoto University) (all to G.O.). This work was also supported by the 10.13039/501100001691JSPS WISE program: “The Graduate Program for Medical Innovation (MIP),” to Y.Y. The funders had no role in study design, decision to publish, or preparation of the manuscript.

## CRediT authorship contribution statement

**Yuki Yamawaki:** Validation, Investigation, Writing – original draft, Writing – review & editing. **Yayoi Wada:** Validation, Investigation, Writing – review & editing. **Sae Matsui:** Validation, Investigation. **Gen Ohtsuki:** Conceptualization, Software, Validation, Investigation, Writing – original draft, Writing – review & editing, Visualization, Supervision, Project administration, Funding acquisition.

## Declaration of competing interest

The authors declare that they have no known competing financial interests or personal relationships that could have appeared to influence the work reported in this paper.
